# Are the non-weight bearing guidelines for the after treatment of calcaneal fractures still decisive? A Dutch survey among orthopaedic and trauma surgeons

**DOI:** 10.1007/s00590-023-03637-4

**Published:** 2023-07-08

**Authors:** Coen Verstappen, Mitchell L. S. Driessen, Pishtiwan H. S. Kalmet, Erik Hermans, Michael J. R. Edwards, Martijn Poeze

**Affiliations:** 1 Department of Surgery, Maastricht University Medical Center+, Maastricht, The Netherlands; 2 Department of Surgery, Radboud University Medical Center, Nijmegen, The Netherlands

**Keywords:** Survey, Displaced intraarticular calcaneal fractures, Trauma patients, Postoperative treatment, Rehabilitation, Permissive weightbearing

## Abstract

**Purpose:**

The current rehabilitation for patients with surgically treated displaced intra-articular calcaneal fractures (DIACFs) consists of non-weightbearing for 8–12 weeks. The purpose of the present survey was to investigate the current pre-, peri- and post-operative practices among Dutch foot and ankle surgeons. Moreover, it aims to analyze whether surgeons comply to the *Arbeitsgemeinschaft für Osteosynthesefragen* (AO) guidelines and which decision criteria were used in the determination of the start of weightbearing.

**Methods:**

A survey was distributed among Dutch trauma and orthopaedic surgeons to determine the most common practices in postoperative weightbearing in patients with DIACFs.

**Results:**

75 surgeons responded to the survey. 33% of the respondents adhered to the AO guidelines. 4% of the respondents strictly followed non-weightbearing guidelines, while 96% interpret the AO guidelines or their local protocol freely, in any frequency. When respondents tended to deviate from the AO guidelines or local protocol, a good patients’ compliance to therapy was expected. 83% of the respondents started weightbearing on the fracture, based on reported patient complaints. 87% of the respondents did not see any relation between early weightbearing and the occurrence of complications, including loosening of osteosynthesis materials.

**Conclusion:**

This study demonstrates that there is limited consensus on the rehabilitation for DIACFs. Moreover, it shows that most surgeons are inclined to interpret the current (AO) guideline or their own local protocol freely. New guidelines, supported with well-founded literature, could help surgeons in a more appropriate daily practice in weightbearing for the rehabilitation of calcaneal fractures.

**Supplementary Information:**

The online version contains supplementary material available at 10.1007/s00590-023-03637-4.

## Introduction

The annual incidence of calcaneal fractures is approximately 11.5 per 100,000 patients and occurs 2.4 times more frequently in males comparing to females [1]. Operative treatment is often needed in case of displaced intraarticular calcaneal fractures (DIACFs), which are generally classified by the Sanders classification [2]. Even after successful operative treatment, long rehabilitation is required with major impact on activities of daily living, quality of life and socio-economic aspects [3, 4]. Anatomic surgical restoration does not prevent gait disturbances or persistent foot pain, yet it is thought to optimize functional outcome [5].

The last few decades new surgical techniques have been developed to improve functional outcomes. Percutaneous techniques (PT) (i.e., Forgon and Zadravecz) have been proposed to aim at lowering soft tissue compromise and improving overall outcomes [6, 7]. While the Extensile Lateral Approach (ELA), remains the most widely used approach, the Sinus Tarsi Approach (STA) is gaining ground [8].

According to the *Arbeitsgemeinschaft für Osteosynthesefragen* (German: “working group for bone fusion issues”) Principles of Fracture Management (‘AO guidelines’), the current aftercare protocol for patients with DIACFs consists of 8 to 12 weeks of non-weightbearing (NWB) followed by partial weightbearing with a weekly increase of 25% in weight loading [9]. These guidelines have been the standard for decades, even though the positive effects of early weightbearing on fracture healing and maintaining muscles and bone mass are well known [10]. Therefore, it can be questioned if the guidelines should be interpreted more broadly.

The purpose of this survey is to investigate pre-, peri- and post-operative practices among Dutch trauma and orthopaedic surgeons, regarding patients with DIACFs. Moreover, it aims to analyze whether surgeons comply to the current non-weight bearing guidelines and which criteria were used to decide when and at what level to start weightbearing after surgically treated DIACFs.

## Materials and methods

A web-based survey was developed using online software (http://www.surveymonkey.nl) and was distributed among Dutch trauma and orthopaedic surgeons.

All hospitals in the Netherlands were contacted directly through phone and e-mail in the period of May 2021 to October 2021 to find out whom of the trauma and orthopaedic surgeons are involved in lower extremity fracture management. The survey was published in the newsletter of the Dutch Trauma Society (NVT) and was posted as a news item on their website. Finally, the survey was brought under attention by distributing flyers at the Dutch trauma congress (‘Traumadagen’) in 2021. The survey consists of 23 multiple choice and 7 open questions as shown in Supplementary Information 1, a summary of the survey is shown in Table 1. The NVT has 597 members, of which it was estimated that around 130 surgeons are regularly involved in treating calcaneal fractures surgically.

**Table 1:** Key points, survey on pre-, peri- and postoperative practices regarding surgically treated calcaneal fractures

Preoperative assessment
Demographics and work experience
Incidence of fracture care
Preferences on type of treatment
Radiographical preferences
(Assumptions on) time-to-surgery
Perioperative assessment
Use of fluoroscopy
Use of antibiotics
Assessment and criteria on successful treatment
Postoperative assessment
Radiographical preferences
Postoperative hospital admission
Assumptions on postoperative weight bearing and rehabilitation
Complications
Closing questions

### Statistical analysis

Statistical analysis was performed using IBM SPSS Statistics, Version 28.0, Armonk, NY. Descriptive statistics were used to describe the demographic data and baseline characteristics of the entire survey. Results are presented as mean ± standard deviation (SD) or as frequencies and percentages, unless indicated otherwise.

## Results

A total of 75 surgeons responded to the survey. 54 (72%) were trauma surgeons and 19 (25%) were orthopaedic surgeons. Two respondents (3%) were residents.

All respondents were active as surgeons with a mean experience time in treating calcaneal fractures of 14.1 years. 44% of the respondents did an additional fellowship in foot and ankle surgery. 23 respondents (31%) worked in a level-I, 39 respondents (52%) in a level-II and 13 respondents (17%) in a level-III trauma center [11].

**Fig. 1:**
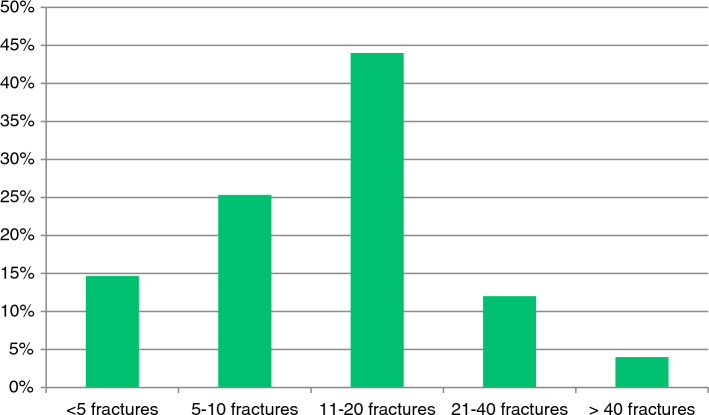
Estimated percentage of number of surgically treated calcaneal fractures per center per annum

30 respondents (40%) indicated that their center treated less than 10 fractures operatively, in 42 centers (56%) 11–40 fractures were operatively treated, and in 3 centers (4%) more than 40 calcaneal fractures were treated operatively per year, as shown Fig. 1.

### Preoperative assessment (n = 75)

Most of the respondents classified calcaneal fractures by evaluating the subtalar joint (fracture gap and step-off) on a CT-scan (84%). 71% did so by using the Sanders classification [2]. One respondent used the Zwipp classification, 26% of the surgeons used the Essex-Lopresti classification [12, 13].

9 out of 10 respondents preferred to treat non-displaced fractures (Sanders type I) conservatively. 61% to 77% of the respondents were very likely to treat a Sanders type II or III fracture by using ELA or STA. STA was more preferred than the ELA. Between 13 and 23% were very likely to consider the use of PT.

The treatment of Sanders type IV calcaneal fractures showed the most variation in the preferred treatment. 34% of the respondents inclined towards the use of the ELA as treatment of choice. To a lesser extent the STA (20%), conservative treatment (16%), primary arthrodesis (16%) and PT (14%) were chosen.

According to the respondents the ideal time from trauma to surgery (TTS) would be 9–10 days. The self-estimated mean TTS was 9.9 days.

### Perioperative assessment (n = 31)

Independent of the type of surgery or fracture, almost 66% of the respondents used one fluoroscope, 16% of the respondents used two. Another 16% used a 3D technique. All respondents provided their patients preoperative antibiotic prophylaxis.

Almost all respondents (94%) considered a congruent subtalar joint or a congruent angle of Böhler (87%) as a marker for successful reposition [14]. Approximately half of them (48%) applied the difference in pre- and postoperative angle of Gissane to describe the reduction [13].

### Postoperative assessment (n = 31)

**Fig. 2:**
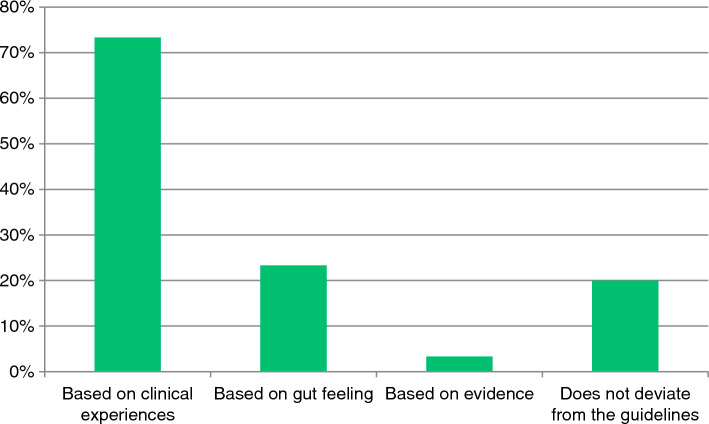
Motivation for deviating from local protocol

The estimated mean postoperative hospital admission time was 2.2 days. Generally, 33% of the respondents adhered to the current non-weight bearing guidelines (8–12 weeks NWB). 47% of the respondents started weightbearing after wound healing. 4% of the surgeons strictly followed their local (non-weightbearing) guidelines, while 96% of the respondents interpret their local protocol more freely, in any frequency. If so, a presumed good patients’ compliance to therapy is expected by 73% of the respondents, shown in Fig. 2.

**Fig. 3:**
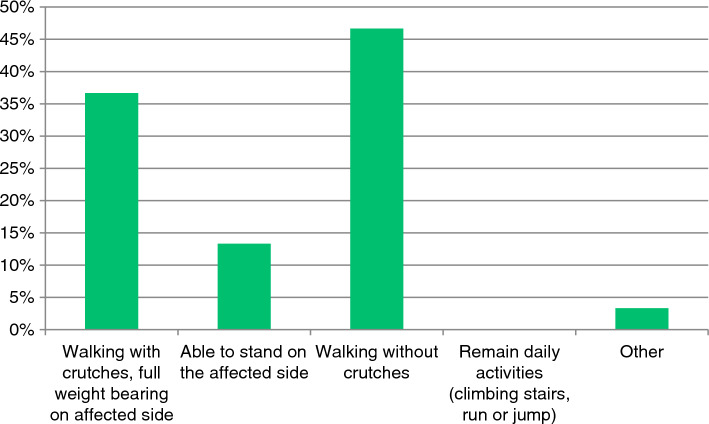
Definition of ‘full weight bearing’

83% starts weightbearing with increasing the actual load weekly, based on reported patient complaints. As shown in Fig. 3, full weightbearing (FWB) was described as walking without crutches or walking with one crutch, but with FWB on the affected side by 83%. 13% described FWB as the ability to stand on the affected side. 87% reported not to see any relation between early weightbearing and the occurrence of complications, including loosening of osteosynthesis materials. All respondents requested their patients to return to the outpatient clinic for the first check-up, two weeks postoperatively.

**Fig. 4:**
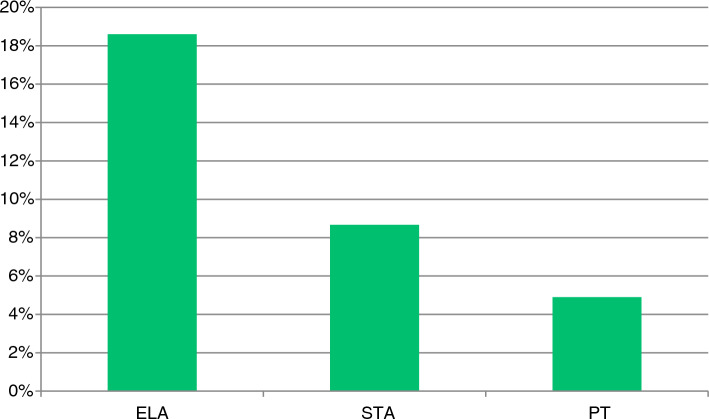
Surgeon estimated complication rate per operation technique. Abbreviations: ELA = Extended Lateral Approach, STA = Sinus Tarsi Approach, PT = percutaneous techniques

Figure 4 shows that respondents expect the ELA to be most likely to cause complications (19%). While STA and PT are thought to be the cause of complications in 9% and 5%.

## Discussion

The current study investigated the current state of pre-, peri- and postoperative practice of calcaneal fractures and the start of aftercare involving weightbearing among Dutch (orthopaedic) trauma surgeons, specialized in foot- and ankle fracture management.

### Preoperative assessment

This study illustrated a consensus on the preferred TTS. The desired TTS in this study is comparable to international literature [15]. Nevertheless, the ideal TTS for DIACFs remains a debate in many studies [16–18]. Although no golden standard exists, to prevent soft tissue complications, the ‘wrinkle test’ is performed to determine the decrease of swelling.

Although in recent literature it was stated that radiological measurements should not be considered to be guidance in the postoperative period, this study shows that the Sanders classification and evaluation of the subtalar joint are mostly used as methods to classify DIACFs. Literature confirms the popularity of the Sanders classification as the most well-known and used system for describing DIACFs [19, 20].

Two-third of the foot and ankle surgeons in this study consider ORIF as preferred technique in treating Sanders type II fractures. There was a mild preference for STA over ELA. In 2008, a study conducted by Schepers et al. showed that the majority of the patients were treated with ORIF (i.e., ELA and STA 46%), conservatively (39%) or with percutaneous screw fixation (10%) [21]. In contrast to our survey, the study by Schepers et al. did not differentiate between more or less severely dislocated fractures by using the Sanders classification. A recent systematic review concluded that STA may be the preferred approach in the operative treatment of patients with displaced Sanders type II and III DIACFs in order to reduce wound healing complications, time-to-surgery and operative time and shows a similar restoration of calcaneal anatomy [15]. Although better clinical outcomes favoring STA seem to be achieved, recent literature showed no significant difference in the cost-effectiveness of ELA versus STA (ELA $8766.8 ± 2835.2/QALY, STA $7914.9 ± 1822.0/QALY respectively) [22].

Between 13 and 23% of the surgeons would consider PT as preferred technique in treating Sanders type II and III. Another 13% would consider PT for Sanders type IV. This is a rise in numbers, compared to a national survey performed by Schepers et al. in 2008 (10%), however, in that study no differentiation in Sanders type was made [21]. Although these numbers are rising in the Netherlands, PT might be undervalued. A recent study from colleagues of our research group comparing ELA and PT in DIACFs with a follow-up of 20 years, showed similarity in restoring Böhlers angle, with similar functional outcomes [23].

Three decades ago, Sanders et al. suggested that primary subtalar arthrodesis (PSTA) has advantages in treating patients with DIACFs, type IV [24]. Since then, PSTA has been the golden standard. However, a recent, randomized multicenter trial showed no significant difference in the treatment of type IV fractures between ORIF and ORIF combined with PSTA [4]. The results in this survey show that surgeons in the Netherlands are indecisive as it comes to the treatment of Sanders type IV. Only a minority of respondents choses for PSTA as treatment of choice.

### Perioperative assessment

This study showed that almost all surgeons use fluoroscopy during the operation, while 32% of the surgeons used a 3D evaluation technique. The effect of a 3D technique remains unclear as in recent literature it was stated that there is no benefit for the use of intraoperative 3D fluoroscopy with regard to postoperative complications, quality of life, functional outcome, or posttraumatic osteoarthritis [25]. Yet, the use of the 3D technique leads to a significant increase of 18% in operative time [25].

### Postoperative assessment

Our survey showed that 90% of the respondents does not think there is an association between early weightbearing and the occurrence of complications. The estimated complication rate for the ELA is in line with a retrospective case series, that found wound infection rate of 25% [26]. The estimated complication rate for the STA corresponds well to a review with a complication rate of 9% [27]. For PT the estimated complication rate was 5% and is not comparable to literature, however heterogeneity in definitions of complications make it difficult to compare the results [28].

Secondly, this study aimed to illustrate the compliance to the current non-weight bearing guidelines or the local protocol. For more than fifty years, the AO advises 8 to 12 weeks NWB for the aftercare of patients with DIACFs [9]. It is generally assumed that trauma and orthopedic surgeons instruct patients restricted weightbearing during rehabilitation. However, the results from our survey show that a large group of surgeons in the Netherlands is inclined to start weightbearing earlier than the proposed 8 to 12 weeks. When choosing for earlier weightbearing, surgeons are likely to increase the loading on the injured foot step-by-step, based on complaints of the patient. It seems to be that the paradigm is changing, as a the survey from Schepers et al. fifteen years ago, reported a mean period of NWB of 9 weeks [21]. On the other hand, a large analysis of aftercare protocols in foot and ankle fractures stated that weightbearing in calcaneal fractures still seemed to be allowed very cautiously. The analysis shows that almost half of the protocols recommend partial weightbearing only at postoperative week six, the other half of the protocols recommended 50% weightbearing after operative care at week six [29]. It represents the lack of knowledge on the comparison of different aftercare protocols after surgically treated DIACFs.

Based on our findings we conclude that there currently is no consensus whether to restrict patients in weightbearing for 6, or 8 to 12 weeks. More and more studies show that permissive weightbearing reduces time to FWB, return to work, and thus lowers the socioeconomic impact, while negative effects on patients reported quality of life, pain or complication rates are rarely reported [30]. A recent review states multiple positive effects in early weightbearing while aftercare from operatively treated DIACFs [31]. Although many studies suggested the benefits of early weightbearing, yet there currently is no prospective literature available that compares operatively treated DIACFs followed by 8 to 12 weeks of restrictive weightbearing with permissive weightbearing, let alone subdivided for different fracture types or approaches. On the other hand, there is no recent evidence that demonstrates the advantages of restrictive weightbearing. Unfortunately, this study does not answer relations between complications and (early) weightbearing.

### Strengths and limitations

The strength of this survey study is that it gives a good overview about the national expert opinion of trauma and orthopaedic surgeons involved in lower extremity fracture management. Moreover, as this study has some similarities with a survey study conducted by Schepers et al. (2008), it offers the opportunity to assess the general trend over time [21].

This study also has some limitations. First, this study is a national study. Although the Netherlands is a small country, it is well-known for its progressive healthcare policies, when it comes to outcomes, access to healthcare and drugs [32]. Therefore, the results of this study are limitedly generalizable to other European, North American, and Asian countries.

The results in the current study are survey-based. Therefore, the results are subject to recall bias. To prevent the occurrence of recall error as much as possible, questions about situations in the distant past were excluded. Nevertheless, it has played a major role in the results of the current study. Besides, because this study is a Likert scaled survey, it is subject to information loss [33].

The overall response rate of the survey was lower compared to the study performed by Schepers et al. However, in a Canadian study, a response rate of 29,6% was reported under general surgeons, which is slightly lower than the response rate of the current survey [34]. Moreover, our response rate is in line with another survey on calcaneal fractures [35]. Lastly, the study faced with incompleteness of data, as only 40% completed the entire questionnaire. This might indicate that the survey was too long. Paradoxically, reducing the number of questions might have led to a decrease in the amount of data gathered from Dutch foot- and ankle surgeons.

## Conclusion

This survey assessed the expert opinion on the treatment and aftercare of DIACFs in the Netherlands. We have demonstrated that there is limited consensus on the aftercare of calcaneal fractures. The results of the current survey might demonstrate that surgeons in the Netherlands tend to be liberal in using the international guidelines although scientific substantiation lacks. Moreover, it shows that the majority of surgeons are inclined to interpret the current non-weight bearing guidelines or their own local protocol freely. New guidelines, supported with well-founded literature, could help surgeons in a more appropriate daily practice in weightbearing for the aftercare of calcaneal fractures.

### Supplementary Information

Below is the link to the electronic supplementary material.

Supplementary file1 (DOCX 25 kb)

## References

[1] Mitchell M, McKinley J, Robinson C (2009). The epidemiology of calcaneal fractures. Foot.

[2] Sanders R, Fortin P, DiPasquale T, Walling A (1993). Operative treatment in 120 displaced intraarticular calcaneal fractures: results using a prognostic computed tomography scan classification. Clin Orthop Relat Res.

[3] Sanders R, Vaupel ZM, Erdogan M, Downes K (2014). Operative treatment of displaced intraarticular calcaneal fractures: long-term (10–20 Years) results in 108 fractures using a prognostic CT classification. J Orthop Trauma.

[4] Buckley R, Leighton R, Sanders D, Poon J, Coles CP, Stephen D (2014). Open reduction and internal fixation compared with ORIF and primary subtalar arthrodesis for treatment of sanders type IV calcaneal fractures: a randomized multicenter* trial. J Orthop Trauma.

[5] van Hoeve S, de Vos J, Verbruggen JP, Willems P, Meijer K, Poeze M (2015). Gait analysis and functional outcome after calcaneal fracture. J Bone Joint Surg Am.

[6] Li LH, Guo YZ, Wang H, Sang QH, Zhang JZ, Liu Z (2016). Less wound complications of a sinus tarsi approach compared to an extended lateral approach for the treatment of displaced intraarticular calcaneal fracture A randomized clinical trial in 64 patients. Medicine.

[7] Forgon M, Zadravecz G (1990) Die Kalkaneus-fraktur. Hefte zur Unfallheilkunde

[8] Khazen G, Rassi CK (2020). Sinus Tarsi approach for calcaneal fractures: the new gold standard?. Foot Ankle Clin.

[9] Buckley RE, Moran CG, Apivatthakakul T (2017) AO principles of fracture management: Vol. 1: principles, Vol. 2: specific fractures. Thieme

[10] Kalmet PH, Meys G, Evers SM, Seelen HA, Hustinx P, Janzing H (2018). Permissive weight bearing in trauma patients with fracture of the lower extremities: prospective multicenter comparative cohort study. BMC Surg.

[11] Landelijk Netwerk Acute Zorg (2020) Levelcriteria Traumachirurgie van de Nederlandse Vereniging voor Traumachirurgie

[12] Zwipp H, Tscherne H, Wülker N, Grote R (1989). Intra-articular fracture of the calcaneus. Classification, assessment and surgical procedures. Der Unfallchirurg..

[13] Essex-Lopresti P (1952). The mechanism, reduction technique, and results in fractures of the os calcis. Br J Surg.

[14] Böhler L (1931). Diagnosis, pathology, and treatment of fractures of the os calcis. JBJS.

[15] Nosewicz TL, Dingemans SA, Backes M, Luitse JSK, Goslings JC, Schepers T (2019). A systematic review and meta-analysis of the sinus tarsi and extended lateral approach in the operative treatment of displaced intra-articular calcaneal fractures. Foot Ankle Surg.

[16] De Boer AS, Van Lieshout EMM, Van ‘t Land F, Misselyn D, Schepers T, Den Hartog D (2018). Soft tissue complications and timing of surgery in patients with a tongue-type displaced intra-articular calcaneal fracture: an international retrospective cohort study. Injury.

[17] Bläsius FM, Stockem LE, Knobe M, Andruszkow H, Hildebrand F, Lichte P (2022). Predictors for wound healing complications and prolonged hospital stay in patients with isolated calcaneal fractures. Eur J Trauma Emerg Surg.

[18] Rammelt S, Sangeorzan BJ, Swords MP (2018). Calcaneal fractures-should we or should we not operate?. Indian J Orthop.

[19] Schepers T, van Lieshout EM, Ginai AZ, Mulder PG, Heetveld MJ, Patka P (2009). Calcaneal fracture classification: a comparative study. J Foot Ankle Surg.

[20] Bulut T, Gursoy M (2021). Are radiological measurements reliable methods in the postoperative follow-up of calcaneal fractures?. Niger J Clin Pract.

[21] Schepers T, van Lieshout EM, van Ginhoven TM, Heetveld MJ, Patka P (2008). Current concepts in the treatment of intra-articular calcaneal fractures: results of a nationwide survey. Int Orthop.

[22] Li Z, Wu X, Zhou H, Xu S, Xiao F, Huang H (2020). Cost-utility analysis of extensile lateral approach versus sinus tarsi approach in Sanders type II/III calcaneus fractures. J Orthop Surg Res.

[23] Driessen MLS, Verstappen C, Poeze M, Edwards M, Biert J, Hermans E (2022). Treatment of displaced intra-articular calcaneal fractures: a single-center experience study with 20 years follow-up. Injury.

[24] Sanders R, Hansen S, McReynolds I (1991) Trauma to the calcaneus and its tendon. Disorders of the Foot and Ankle: Medical and Surgical Management, 2nd edn. Philadelphia: WB Saunders, pp 2326–54

[25] Halm JA, Beerekamp MSH, de Muinck-Keijzer RJ, Beenen LFM, Maas M, Goslings JC (2020). Intraoperative effect of 2D vs 3D fluoroscopy on quality of reduction and patient-related outcome in calcaneal fracture surgery. Foot Ankle Int.

[26] Backes M, Spierings KE, Dingemans SA, Goslings JC, Buckley RE, Schepers T (2017). Evaluation and quantification of geographical differences in wound complication rates following the extended lateral approach in displaced intra-articular calcaneal fractures-a systematic review of the literature. Injury.

[27] Schepers T (2011). The sinus tarsi approach in displaced intra-articular calcaneal fractures: a systematic review. Int Orthop.

[28] Schepers T, Schipper IB, Vogels LM, Ginai AZ, Mulder PG, Heetveld MJ (2007). Percutaneous treatment of displaced intra-articular calcaneal fractures. J Orthop Sci.

[29] Pfeifer CG, Grechenig S, Frankewycz B, Ernstberger A, Nerlich M, Krutsch W (2015). Analysis of 213 currently used rehabilitation protocols in foot and ankle fractures. Injury.

[30] Kalmet PHS, Van Horn YY, Sanduleanu S, Seelen HA, Brink PR, Poeze M (2019). Patient-reported quality of life and pain after permissive weight bearing in surgically treated trauma patients with tibial plateau fractures: a retrospective cohort study. Arch Orthop Trauma Surg.

[31] De Boer AS, Van Lieshout EMM, Van Moolenbroek G, Den Hartog D, Verhofstad MHJ (2018). The effect of time to post-operative weightbearing on functional and clinical outcomes in adults with a displaced intra-articular calcaneal fracture; A systematic review and pooled analysis. Injury.

[32] Schneider EC, Shah A, Doty MM, Tikkanen R, Fields K, Williams R (2021) Mirror, mirror 2021: Reflecting poorly: Health care in the US compared to other high-income countries. Commonwealth Fund

[33] Westland JC (2022). Information loss and bias in likert survey responses. PLoS ONE.

[34] Cunningham CT, Quan H, Hemmelgarn B, Noseworthy T, Beck CA, Dixon E (2015). Exploring physician specialist response rates to web-based surveys. BMC Med Res Methodol.

[35] Pastor T, Gradl G, Klos K, Ganse B, Horst K, Andruszkow H (2016). Displaced intra-articular calcaneal fractures: is there a consensus on treatment in Germany?. Int Orthop.

