# Supplementary material for: Are the non-weight bearing guidelines for the after treatment of calcaneal fractures still decisive? A Dutch survey among orthopaedic and trauma surgeons
Source: Eur J Orthop Surg Traumatol. 2023 Jul 8;34(1):209–16. doi: 10.1007/s00590-023-03637-4 (PMC10771614; doi:10.1007/s00590-023-03637-4)
Supplement: Supplementary file 1 — Supplementary file1 (DOCX 25 kb) [file 590_2023_3637_MOESM1_ESM.docx]

Supplementary Information 1 (SI 1). *Questions survey*.

***30 questions about the treatment and rehabilitation of calcaneal fractures in Dutch hospitals***

Introduction and (demographic) characteristics

1. Are you a trauma surgeon or orthopedic surgeon?
   1. Yes, trauma surgeon
   2. Yes, orthopedic surgeon
   3. No, namely…
      1. *Answer field*
2. How long have you been working in this position since your graduation as surgeon?
   1. *Answer field* … year
3. Demographic data about your hospital
   1. How many beds does your clinic have according to the Ministry of Health, Welfare and Sport.
      - >1000, 'large'
      - 500-1000, 'medium'
      - –500, 'small'
   2. What is the level of trauma center of your hospital?
      - Level 1
      - Level 2
      - Level 3
4. How many calcaneal fractures does your hospital treat per year, both **conservatively** and **surgically**?
   1. Less than 5
   2. 5-10
   3. 11-20
   4. 21-40
   5. More than 40
5. How many **operative** calcaneus fractures does your clinic treat per year?
   1. Less than 5
   2. 5-10
   3. 11-20
   4. 21-40
   5. More than 40
6. Do you have specific expertise in the surgical treatment of calcaneal fractures?
   1. Yes, by experience
   2. Yes, by following a trauma surgery fellowship
   3. Yes, by following a specific foot and ankle surgery fellowship
   4. No
7. How many years of experience do you have in treating calcaneal fractures?
   1. *Answer field* … year.

Motivation for type of treatment

1. Which treatment do you prefer? The following statements:
   1. “I tend to treat **conservatively** in case of:”

*5-Point/Likert Scale*

- - - Extra-articular fractures
    - Intra-articular, non-displaced fractures
    - Displaced intra-articular fractures
    - A normal calcaneus length
    - A normal angle of Böhler
    - More than three comorbidities (e.g. diabetes mellitus, COPD, atrial fibrillation, heart failure…)
    - Bad soft tissue in the operation area
    - Limited patient compliance
    - Smokers
    - Patients with severe mental illness
  1. “I perform percutaneous screw fixation in the following situations / in the following fractures / patients:”

*5-Point/Likert Scale*

- - - Low classified fractures (e.g. Sanders type I-II)
    - Intact posterior facet
    - Minimally modified angle of Böhler
    - Minimally dislocated fracture
    - Elderly patients
    - A poor general condition (ASA III and IV)
    - More than three comorbidities (e.g. diabetes mellitus, COPD, atrial fibrillation, heart failure…)
    - Poor tissue quality
    - A mental illness of the patient
    - Limited patient compliance
    - Subjective parameters of the surgeon
    - Smokers

Preoperative

1. Which **preoperative radiological features** are used in your clinic?

*Tick a box ​​(multiple answers possible)*

- - - Essex-Lopresti classification by X-ray
    - Böhler’s angle using X-ray
    - Böhler’s angle by CT scan
    - Evaluation of the subtalar joint (fracture gap and step-off) by CT scan
    - Number of fracture fragments and degree of discrepancy (assessing the pars anterior, the posterior facet or the calcaneus as a whole) by CT scan
    - Sanders classification by CT scan
    - Zwipp classification by CT scan
    - Different classification system or criteria; namely:

o *Answer field*

1. Which type of treatment do you prefer for the following fractures, by classification? Choose the most appropriate answer.
   1. Sanders type I
   - Conservative
   - Percutaneous screw fixation according to Forgon-Zadravecz
   - ORIF (Extended Lateral Approach)
   - ORIF (Sinus Tarsi Approach)
   - External fixator
   - Reposition via K-wires
   - Primary arthrodesis
   1. Sanders type II
   - Conservative
   - Percutaneous screw fixation according to Forgon-Zadravecz
   - ORIF (Extended Lateral Approach)
   - ORIF (Sinus Tarsi Approach)
   - External fixator
   - Reposition via K-wires
   - Primary arthrodesis
   1. Sanders type III
   - Conservative
   - Percutaneous screw fixation according to Forgon-Zadravecz
   - ORIF (Extended Lateral Approach)
   - ORIF (Sinus Tarsi Approach)
   - External fixator
   - Reposition via K-wires
   - Primary arthrodesis
   1. Sanders type IV
   - Conservative
   - Percutaneous screw fixation according to Forgon-Zadravecz
   - ORIF (Extended Lateral Approach)
   - ORIF (Sinus Tarsi Approach)
   - External fixator
   - Reposition via K-wires
   - Primary arthrodesis
2. What is the estimated time-to-surgery (trauma to surgery) in your hospital?
   1. *Answer field* … days
3. What do you think that the time-to-surgery (trauma to surgery) should be?
   1. *Answer field* … days

Perioperative

1. Do you use screening during surgery?
   1. Yes, I use one X-ray tube
   2. Yes, I use two X-ray tubes
   3. Yes, I use 3D technology
   4. No
2. Do you treat the patient with antibiotics?
   1. Yes, perioperatively only
   2. Yes, both peri- and post-operatively up to 24 hours
   3. Yes, both peri- and post-operatively up to 3 days
   4. Yes, both peri- and post-operatively up to 7 days
   5. Yes, otherwise:

- *Answer field*
  1. No

1. The following factor(s) determine whether my reposition was successful:
   1. A congruent subtalar joint
   2. A restored Böhler’s angle
   3. A restored Gissane’s angle
   4. A restored varus or valgus position of the tubercle
   5. Other, namely…

- *Answer field*

Postoperative

1. What post-operative radiological features are used in your clinic?

*Tick a box ​​(multiple answers possible)*

- Essex-Lopresti classification by X-ray
- Böhler’s angle using X-ray
- Böhler’s angle by CT scan
- Evaluating the subtalar joint (fracture gap and step-off) by CT scan
- Number of fracture fragments and degree of discrepancy (assessing the pars anterior, the posterior facet or the calcaneus as a whole) by CT scan
  - - Different classification system or criteria; namely:

o *Answer field*

Rehabilitation and uncertainties

1. On average, how long do patients stay in your hospital after a surgically treated calcaneus fracture?
   1. *Answer field* … days.
2. What is the standard rehabilitation protocol in your hospital? Choose the answer that best suits the protocol you are using.
   1. By plaster; 6 weeks *non* *weight-bearing*
   2. By plaster; 12 weeks *non weight-bearing*
   3. By plaster until the first outpatient appointment, then guided by the clinic; *permissive weight-bearing*
   4. By walking plaster, with *permissive weight-bearing* after wound healing (2 weeks)
   5. By walking plaster, with *full weight-bearing* after wound healing (2 weeks)
   6. No plaster, with *permissive weight-bearing* after wound healing (2 weeks)
   7. No plaster, with *full weight-bearing* after wound healing (2 weeks)
3. When do you advise patients with surgically treated calcaneus fractures to start weight bearing, and at what percentage do you start?
   1. After *answer field* … weeks, patients can start loading.
   2. With *answer field* … percent weight-bearing.
4. Do you or your colleagues often deviate from the standard follow-up treatment that is used in your hospital?

*5-Point/Likert Scale*

- 1. Very often / often / sometimes / rarely / never

1. If you deviate from the standard rehabilitation protocol, on what factors is this based?
   1. Based on clinical experience
   2. Based on gut feeling
   3. Based on evidence-based medicine
   4. I never or rarely deviate from the standard rehabilitation protocol
2. What criteria do you use to determine whether earlier or later weight-bearing is possible?
   1. Fracture type
   2. Type of osteosynthesis material
   3. The age of the patient
   4. The estimated consolidation via imaging (e.g. degree of callus formation)
   5. Based on patient comorbidities
   6. Based on expected patient compliance
3. How do you define 100% weight-bearing?
   1. Walking with crutches and full load on affected side
   2. Being able to stand on the affected side
   3. Walking without crutches
   4. Having resumed activities such as climbing stairs, running, or jumping
   5. Otherwise, namely:

- *Answer field*

1. In which steps do you increase the load?
   1. Step-by-step increase over a predetermined number of weeks, expressed in kilograms or as a percentage of body weight
   2. Step-by-step increase over a predetermined number of weeks, expressed in the number of kilograms that allow the load according to the patient
   3. Gradual increase in load based on patient complaints
   4. Otherwise, namely:

- *Answer field*

1. When will you see your patients in the outpatient clinic for the first time after treatment?
   1. After 2 weeks
   2. After 4 weeks
   3. After 6 weeks
   4. After 8 weeks
2. What complications do you see in your clinic after the surgical treatment of calcaneus fractures?

*Tick a box ​​(multiple answers possible)*

- 1. Infection
  2. Impaired reposition, compared to per or short postoperatively
  3. Non-union
  4. Mal-union
  5. Decreased range of motion
  6. Otherwise, namely:
- *Answer field*

1. Are any of your chosen complications related to early weight-bearing?
   1. Yes
   2. no
2. What is the percentage of infections and/or wound healing disorders after applying the following surgical treatments for calcaneus fractures, Extended Lateral Approach / Sinus Tarsi Approach / PT?
   1. ELA

- *Answer field* … percent.
  1. STA
- *Answer field* … percent.
  1. PT
- *Answer field* … percent.

1. I miss better tools and guidelines for the postoperative rehabilitation of calcaneus fractures.
   1. Yes
   2. No

Room for comments.

- *Answer field*…
